# Patient choice for high‐volume center radiation impacts head and neck cancer outcome

**DOI:** 10.1002/cam4.1756

**Published:** 2018-09-02

**Authors:** Arash O. Naghavi, Michelle I. Echevarria, Tobin J. Strom, Yazan A. Abuodeh, Puja S. Venkat, Kamran A. Ahmed, Stephanie Demetriou, Jessica M. Frakes, Youngchul Kim, Julie A. Kish, Jeffery S. Russell, Kristen J. Otto, Christine H. Chung, Louis B. Harrison, Andy Trotti, Jimmy J. Caudell

**Affiliations:** ^1^ Department of Radiation Oncology H. Lee Moffitt Cancer Center and Research Institute Tampa Florida; ^2^ Department of Radiation Oncology UT Southwestern Medical Center Dallas Texas; ^3^ Department of Radiation Oncology UCLA Health Los Angeles California; ^4^ Department of Biostatistics and Bioinformatics H. Lee Moffitt Cancer Center and Research Institute Tampa Florida; ^5^ Department of Senior Adult Oncology H. Lee Moffitt Cancer Center and Research Institute Tampa Florida; ^6^ Department of Head and Neck and Endocrine Oncology H. Lee Moffitt Cancer Center and Research Institute Tampa Florida

**Keywords:** expertise, head and neck cancer, high‐volume center, outcome, radiation, radiotherapy

## Abstract

**Background:**

Studies suggest treatment outcomes may vary between high (HVC)‐ and low‐volume centers (LVC). Radiation therapy (RT) for head and neck cancer (HNC) requires weeks of treatment, the inconvenience of which may influence a patient's choice for treatment location. We hypothesized that receipt of RT for HNC at a HVC would influence outcomes compared to patients evaluated at a HVC, but who chose to receive RT at a LVC.

**Methods:**

From 1998 to 2011, 1930 HNC patients were evaluated at a HVC and then treated with RT at either a HVC or LVC. Time‐to‐event outcomes and treatment factors were compared.

**Results:**

Median follow‐up was 34 months. RT was delivered at a HVC for 1368 (71%) patients and at a LVC in 562 (29%). Patients were more likely to choose HVC‐RT if they resided in the HVC's county or required definitive RT (all *P* < 0.001). HVC‐RT was associated with a significant improvement in 3‐year LRC (84% vs 68%), DFS (68% vs 48%), and OS (72% vs 57%) (all *P* < 0.001). On multivariate analysis (MVA), HVC‐RT independently predicted for improved LRC, DFS, and OS (all *P* < 0.05).

**Conclusions:**

In patients evaluated at a HVC, the choice of RT location was primarily influenced by their residing distance from the HVC. HVC‐RT was associated with improvements in LRC, DFS, and OS in HNC. As treatment planning and delivery are technically demanding in HNC, the choice to undergo treatment at a HVC may result in more optimal delivered dose, RT duration, and outcome.

## 1 INTRODUCTION

Technically demanding treatments benefit from expertise. This was initially investigated in surgical procedures, with multiple reports demonstrating improved survival with increasing experience of the surgeon and facility.^1^, ^2^, ^3^ These findings are more pronounced when treating diseases with low incidence, as in the case with pancreatic cancer.^1^ These findings suggest a benefit in centralization of care to high‐volume center (HVC) for complex disease sites.

In 2017, an estimated 49 670 new patients are expected to be diagnosed with head and neck cancer (HNC) in the United States.^4^ Radiotherapy (RT) for HNC, especially intensity‐modulated radiotherapy (IMRT), may have large variations in treatment and management that are dependent on the experience of the facility and physician. Head and neck anatomy is complex, with numerous vital structures that must be identified and protected. Beyond normal anatomy, disease/target identification, nodal coverage, and target dosing can vary by institution, with drastic differences in tumor coverage^5^ and RT treatment delivery (eg, dosing, fractionation, treatment duration).^6^ Unfortunately, some facilities may stray from nationally accepted guidelines, which may affect outcome.^6^, ^7^ Centralized RT facilities, such as HVCs, has the potential to improve HNC outcome when compared to low‐volume centers (LVC), but requires 6‐7 weeks of daily RT, which may be inconvenient for the patient.

Previous studies investigating the relationship between RT treatment facilities and outcome in HNC have yielded mixed results.^6^, ^7^, ^8^, ^9^, ^10^, ^11^, ^12^, ^13^ To best determine the effect HVC‐RT treatment has on HNC outcome, we only analyzed patients that were initially evaluated at our HVC, in hopes of controlling for differences in patient workup, diagnosis, and access to care, which may help elucidate factors influencing a patient's choice for HVC‐RT. In this study, we sought to examine the relationship between patient choice of RT treatment facility (HVC vs LVC) and its effect on outcome. We hypothesized that patients evaluated at a HVC, and chose to pursue treatment at a LVC, had an impact on outcome. We also sought to investigate factors associated with choosing LVC‐RT and treatment factors that may influence outcome based on the facility.

## 2 MATERIALS AND METHODS

### 2.1 Patient population

Our inclusion criteria consisted of pathologically confirmed noncutaneous head and neck squamous cell carcinoma patients evaluated at our HVC, treated with radiotherapy, excluding patients with a prior history of cancer or with distant metastatic disease at the time of diagnosis. After IRB approval, records of 6992 HNC patients evaluated between 1998 and 2011 at our HVC were identified. Of those, 2306 patients were treated with RT for nonmetastatic squamous cell carcinoma of the head and neck. An additional 394 patients were excluded because of a prior diagnosis of cancer, leaving 1930 patients for analysis. All patients were evaluated at a HVC and subsequently underwent RT at either a HVC or a LVC. LVCs were defined as nonsatellite facilities that participated in the same cancer registry as our institution. Patient demographics, tumor, treatment information, and outcomes were abstracted from the chart and from cancer registry data.

### 2.2 Determining HVC

Based on the 2010 cancer statistics data, 3.22% of all cancers diagnosed were HNC (49 260 cases).^14^, ^15^ Our state constitutes 7% of the national incidence of all new cancers (107 000 cases), which would be an estimated annual incidence of 3448 new HNCs.^14^ To estimate the annual incidence of our region, we used the population at risk in our state, defined as 18 years of age or older, estimated at 14.8 million people (http://www.census.gov). This gives us a 23.3 HNC incidence per 100 000 people at risk in our state. There are 3.1 million people at risk in our region, which equates to approximately 720 new HNC cases annually (http://www.census.gov). About two‐thirds of HNC patients undergo curative treatment with radiation,^16^ which is 475 patients per year. In 2010, our institution treated 192 new nonmetastatic HNC patients with radiation, accounting for 40.4% of the region's estimated HNC population. Our cohort of patients resides within a quantifiable set of counties, containing over 30 nonsatellite radiation facilities,^17^ each presumably averaging 2% of the remaining HNC cases treated. After stratifying centers into three equal tiers based on the patient volume treated, “high‐volume centers” have been historically defined as the locations responsible for treating the top one‐third of the total patient volume.^9^, ^18^ By this definition, our facility is the only HVC in our cohort.

### 2.3 Statistical method

Demographic and clinical characteristics of HVC and LVC groups were compared using Pearson's chi‐square test for association with categorical variables or Wilcoxon rank‐sum test for difference of continuously observed variables as appropriate. Variables associated with effects on dichotomous outcomes (*P < *0.1) in univariate analysis (UVA) underwent multivariable logistic regression (MVA) to identify factors that were independently associated with the location of RT (HVC vs LVC). Time‐to‐event outcomes were defined as time duration from the date of first treatment to the date of last follow‐up. Locoregional control (LRC) was defined as the time from start of treatment to first local or regional recurrence. Disease‐free survival (DFS) was defined as the time from start of treatment to first local, regional, distant recurrence, or death from any cause. Overall survival (OS) was defined from the start of treatment to death or last patient contact. LRC, DFS, and OS distributions were estimated using Kaplan‐Meier method and were compared via log‐rank test. Variables with marginally significant effect (*P < *0.1) on univariate analysis were included in a multivariable Cox proportional hazard regression analysis, to evaluate their effects on survival hazard risk considering their dependency. Two‐sided *P*‐values and the level of significance of 0.05 were used for statistical analyses, and all analyses were performed using SPSS v 22 (IBM, Armonk, NY).

Pretreatment characteristics evaluated for their association between HVC and LVC groups include the following: follow‐up (months), age (years), gender, race, Spanish ethnicity, county of residence, tumor grade, tobacco use, HPV/p‐16 status, primary disease site, AJCC TNM, and group staging (Table 1). Treatment characteristics compared between HVC and LVC groups include the following: duration of RT treatment (≤7 vs 7 weeks), RT dose for definitive/adjuvant cases (≥66/60 Gy vs <66/60 Gy), use of IMRT, chemotherapy use, diagnosis to treatment initiation (≤45 vs >45 days), and type of radiation (definitive vs adjuvant RT) for the overall cohort (Table 3). In addition, when comparing the differences between HVC and LVC by treatment modality (adjuvant and definitive RT), additional treatment factors compared include the following: location of surgery (HVC vs LVC), surgery to postoperative radiotherapy (TTPORT>7 vs ≤7 weeks), and package time (surgery to completion of postoperative radiation: <100 vs ≥100 days) (Tables 5 and 6). These factors were also evaluated for their association with time‐to‐event outcome for the entire cohort (Table 2) and individually for each treatment type (adjuvant and definitive RT) (Table 4). Variables on univariate analysis that was trending (*P* < 0.1) in each table were subsequently included in the multivariate analysis with variables excluded have a *P*‐value denoted with a dash (“—”) or an asterisks (“*”) on the variable, otherwise variables on the table were included on MVA.

**Table 1:** Comparison of pretreatment characteristics for entire cohort

Comparison of patient and tumor characteristics	Total	HVC	LVC	MVA	
	Median (range)	Median (range)	Median (range)	*P* value	OR (95%CI)
Follow‐up^a^ (mo)	34 (0‐188)	35 (2‐188)	28 (0‐181)		
Age (y)	59 (20‐95)	58 (20‐95)	61 (27‐90)	0.043	0.99 (0.97‐1)
	**Total**	**HVC**	**LVC**	**MVA**	
	**n (%)**	**n (%)**	**n (%)**	***P*** **value**	**OR (95%CI)**
Gender					
Female	425 (22)	286 (21)	139 (25)	0.4	—
Male	1505 (78)	1082 (79)	423 (75)		
Race^a^					
White	1778 (92)	1257 (92)	521 (93)	—	—
Black	82 (4)	56 (4)	26 (5)		
Asian	20 (1)	16 (1)	4 (1)		
Other	45 (2)	36 (3)	9 (2)		
Unknown race	5 (0)	3 (0)	2 (0)		
Spanish ethnicity^a^					
Non‐Spanish	1801 (93)	1271 (93)	530 (94)	—	—
Spanish	120 (6)	90 (7)	30 (5)		
Unknown ethnicity	9 (1)	7 (1)	2 (0)		
Residence					
Elsewhere	1327 (69)	799 (58)	528 (94)	<0.001	17.63 (10.8‐28.77)
County of HVC	603 (31)	569 (42)	34 (6)		
Tumor grade					
Well/Modera diff	986 (51)	661 (48)	325 (58)	0.312	—
Poor/Undiff	621 (32)	440 (32)	181 (32)		
Unknown grade	323 (17)	267 (20)	56 (10)		
Tobacco use					
Current	820 (43)	545 (40)	275 (49)	0.067	—
Former	724 (38)	529 (39)	195 (35)		
Never	219 (11)	188 (14)	31 (6)		
Unknown use	167 (9)	106 (8)	61 (11)		
HPV/P16 status					
HPV (−)	31 (2)	29 (2)	2 (0)	<0.001	REF
HPV (+)	242 (13)	231 (17)	11 (2)	0.397	—
Unknown HPV status	1657 (86)	1108 (81)	549 (98)	0.005	0.09 (0.02‐0.48)
Primary site					
Oral cavity/LIPS	459 (24)	208 (15)	251 (45)	0.032	REF
Oropharynx	843 (44)	696 (51)	147 (26)	0.078	1.56 (0.95‐2.56)
Larynx	345 (18)	259 (19)	86 (15)	0.015	1.85 (1.13‐3.03)
Hypopharynx	84 (4)	61 (5)	23 (4)	0.985	—
Nasopharynx	80 (4)	70 (5)	10 (2)	0.102	2.25 (0.85‐5.94)
Nasal cavity/sinus	71 (4)	47 (3)	24 (4)	0.003	3.37 (1.5‐7.57)
Salivary	23 (1)	11 (1)	12 (2)	0.471	—
Unknown primary	25 (1)	16 (1)	9 (2)	0.513	—
AJCC stage^a^					
I	118 (6)	91 (7)	27 (5)	—	—
II	182 (9)	136 (10)	46 (8)		
III	337 (18)	245 (18)	92 (16)		
IV	1293 (67)	896 (66)	397 (71)		
Tumor stage					
T1	397 (21)	305 (22)	92 (16)	0.388	—
T2	586 (30)	430 (31)	156 (28)		
T3	402 (21)	308 (23)	94 (17)		
T4	516 (27)	306 (22)	210 (37)		
T0/TX	29 (2)	19 (1)	10 (2)		
Nodal stage					
N0	557 (29)	386 (28)	171 (30)	0.084	—
N1	303 (16)	199 (15)	104 (19)		
N2	977 (51)	716 (52)	261 (46)		
N3	93 (5)	67 (5)	26 (5)		

95%CI, 95% confidence interval; HVC, high‐volume center; HPV, human papillomavirus; LVC, low‐volume center; MVA, multivariate analysis; n, number of patients; OR, odds ratio; REF, reference variable.

a Not included on MVA.

## 3 RESULTS

### 3.1 Study characteristics

A total of 1930 patients were analyzed, of which 71% (n = 1368) were treated with RT at a HVC and 29% (n = 562) at a LVC. The median follow‐up for all patients was 34 months. Patient and tumor characteristics are summarized in Table 1. Patient characteristics were relatively well balanced by treatment location, except for county of residence (*P < *0.001), known HPV/p16 status (*P < *0.001), and primary site (*P = *0.032). Patients that live outside of the HVC county were more likely to choose RT at a LVC (vs HVC: 94% vs 58%). Treatment of the oral cavity was more common at a LVC (vs HVC: 45% vs 15%, *P* < 0.001). On logistic regression multivariate analysis, RT at a HVC was associated with residence in the HVC county (OR 17.63, 95%CI 10.8‐28.77, *P < *0.001) and less likely to have unknown HPV status (OR 0.09, 95%CI 0.02‐0.48, *P = *0.005), or oral cavity site treated (*P = *0.032) (Table 1).

### 3.2 Outcomes overall

**Figure 1:**
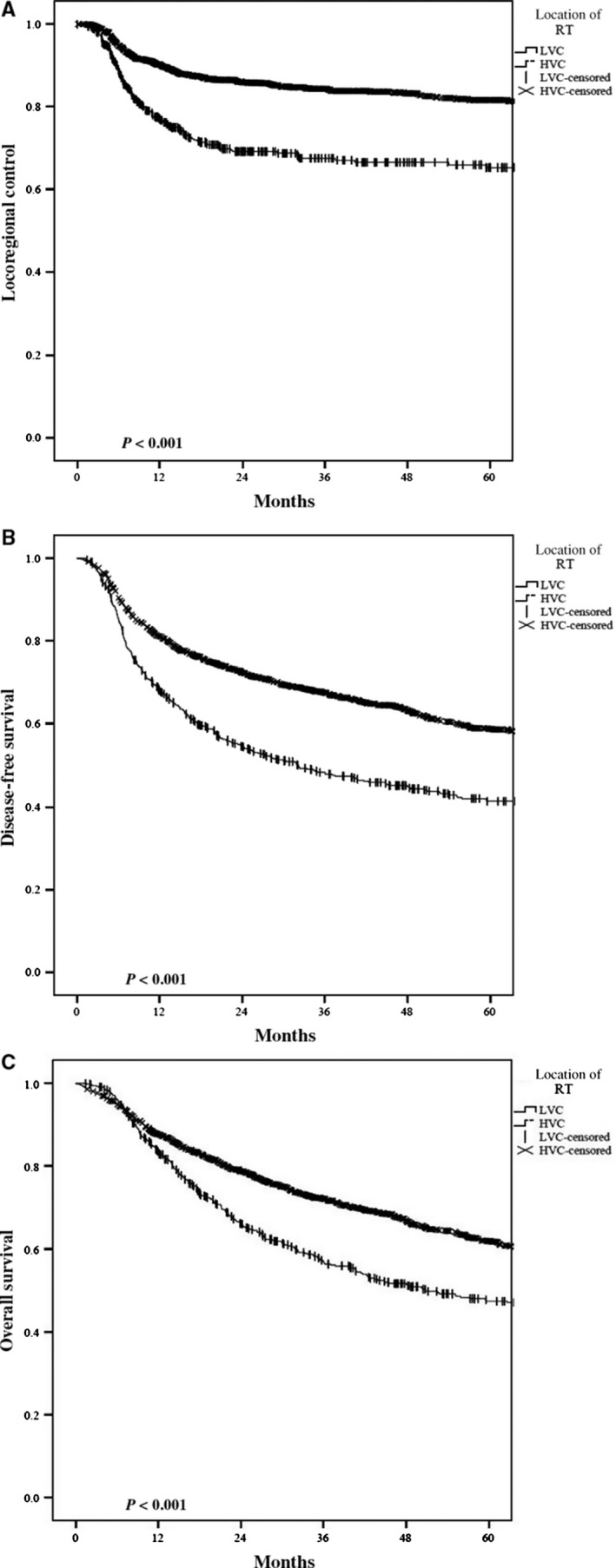
Kaplan‐Meier plots comparing High‐ vs Low‐volume center radiation in regard to (A) locoregional control, (B) disease‐free survival, and (C) overall survival

For the entire cohort, the overall 3‐year LRC, DFS, and OS were 80%, 62%, and 68% respectively. Radiotherapy at a HVC resulted in an absolute 3‐year LRC benefit of 16% (84% vs 68%, *P* < 0.001) (Figure 1A). This benefit at 3 years was also seen with DFS (68% vs 48%) and OS (72% vs 57%) (*P < *0.001) (Figure 1B,C). On multivariate analysis, radiotherapy at a HVC remained independently associated with an improved LRC (HR 0.57, CI95% 0.44‐0.72, *P* < 0.001), DFS (HR 0.78, 95% 0.63‐0.95, *P* = 0.013), and OS (HR 0.69 95%CI 0.53‐0.9, *P* < 0.005) (Table 2).

**Table 2:** Predictors of locoregional control (LRC), disease‐free survival (DFS), and overall survival (OS) on multivariate analysis (MVA) of entire cohort

Multivariate analysis of outcomes	LRC		DFS		OS	
	MVA (*P*)	HR (95% CI)	MVA (*P*)	HR (95% CI)	MVA (*P*)	HR (95% CI)
Location of RT						
HVC	<0.001	0.57 (0.44‐0.72)	0.013	0.78 (0.63‐0.95)	0.898	—
LVC						
Residence						
Elsewhere	0.203	—	<0.001	1.32 (1.13‐1.54)	<0.001	1.44 (1.24‐1.68)
County of HVC						
Tobacco use						
Current	0.246	—	<0.001	REF	<0.001	REF
Former			0.013	0.83 (0.71‐0.96)	<0.001	0.75 (0.64‐0.88)
Never			0.001	0.59 (0.44‐0.8)	<0.001	0.45 (0.32‐0.63)
Unknown use			0.001	0.66 (0.51‐0.84)	0.001	0.65 (0.5‐0.83)
HPV/P16 status						
HPV (−)	<0.001	REF	0.001	REF	<0.001	—
HPV (+)	0.001	0.16 (0.06‐0.47)	0.002	0.35 (0.18‐0.68)	<0.001	0.22 (0.11‐0.48)
Unknown HPV status	0.314	—	0.162	—	0.16	
Primary site						
Oral cavity/LIPS	<0.001	REF	<0.001	REF	<0.001	REF
Oropharynx	<0.001	0.27 (0.19‐0.39)	<0.001	0.44 (0.34‐0.56)	<0.001	0.46 (0.36‐0.6)
Larynx	<0.001	0.32 (0.22‐0.47)	<0.001	0.56 (0.44‐0.7)	<0.001	0.61 (0.48‐0.78)
Hypopharynx	0.012	0.51 (0.3‐0.86)	0.429	—	0.534	—
Nasopharynx	<0.001	0.16 (0.08‐0.33)	<0.001	0.42 (0.28‐0.64)	<0.001	0.43 (0.28‐0.66)
Nasal cavity/sinus	0.089	0.63 (0.38‐1.07)	0.266	—	0.088	0.69 (0.45‐1.06)
Salivary	0.989	—	0.506	—	0.093	1.52 (0.93‐2.48)
Unknown primary	0.937	—	0.665	—	0.345	—
Tumor stage						
T1	<0.001	REF	<0.001	REF	<0.001	REF
T2	0.278	—	0.001	1.46 (1.16‐1.85)	0.001	1.52 (1.18‐1.94)
T3	0.027	1.55 (1.05‐2.29)	<0.001	1.84 (1.45‐2.35)	<0.001	1.96 (1.52‐2.53)
T4	<0.001	2.36 (1.63‐3.41)	<0.001	2.47 (1.95‐3.12)	<0.001	2.72 (2.12‐3.49)
T0/TX	0.935	—	0.978	—	0.997	—
Nodal stage						
N0	0.236	—	<0.001	REF	<0.001	REF
N1	0.716		0.116	—	0.009	1.35 (1.08‐1.7)
N2	0.47		<0.001	1.52 (1.28‐1.81)	<0.001	1.69 (1.41‐2.03)
N3	0.105		<0.001	2.63 (1.93‐3.59)	<0.001	3.09 (2.26‐4.23)
Chemotherapy						
Chemo (+)	0.876	—	0.221	—	0.446	—
Chemo (−)						
RT duration (wk)						
≤7	0.094	REF	0.004	REF	0.001	REF
>7	0.081	1.22 (0.98‐1.53)	0.001	1.27 (1.1‐1.47)	0.001	1.3 (1.12‐1.5)
Unknown duration	0.403		0.318	—	0.022	1.6 (1.07‐2.38)
IMRT						
IMRT (−)	0.242	—	0.011	REF	0.103	—
IMRT (+)			0.003	0.77 (0.65‐0.91)		
Unknown IMRT status			0.154	—		
Total RT dose (NCCN)						
<60/66 Gy	0.71	—	0.135	—	<0.001	REF
≥60/66 Gy					<0.001	0.58 (0.46‐0.74)
Unknown dose					0.117	—
DTI (d)						
46+	0.56	—	0.28	—	0.368	—
≤45						
Age (y)	0.139	—	<0.001	1.02 (1.02‐1.03)	<0.001	1.03 (1.02‐1.04)

DFS, disease‐free survival; DTI, diagnosis to treatment initiation; HVC, high‐volume center; HPV, human papillomavirus; IMRT, intensity‐modulated radiotherapy; LRC, locoregional control; LVC, low‐volume center; MVA, multivariate analysis; OS, overall survival; RT, radiation.

Prolonged RT duration >7 weeks was independently associated with a detriment in LRC (>7 weeks, HR 1.22, 95%CI 0.98‐1.53, *P* = 0.081), DFS (HR 1.27, 95%CI 1.1‐1.47, *P* = 0.001), and OS (HR 1.3 95%CI 1.12‐1.5, *P* = 0.001). Although RT dose (definitive RT ≥66 Gy and adjuvant RT ≥60 Gy), IMRT use, and diagnosis to treatment (≤45 days) predicted for a benefit in LRC, DFS, and OS on univariate analysis, none of these continued to predict for outcome on MVA.

On MVA, HVC‐RT patients were four times more likely to receive adequate RT dose (definitive RT ≥66 Gy or adjuvant RT ≥60 Gy, OR 4.1 CI95% 2.4‐7, *P* < 0.001), and three times less likely to have prolonged RT treatment duration (>7 weeks, OR 0.31 CI95% 0.22‐0.43, *P* < 0.001), when compared to LVC (Table 3). HVC‐RT patients were also more likely treated with definitive RT (74% vs 37%, OR 3.3 CI95% 2.1‐5, *P* < 0.001) and concomitant chemotherapy (69% vs 42%, OR 2.8 CI95% 1.9‐4.1, *P* < 0.001).

**Table 3:** Comparison of treatment characteristics

Treatment characteristics	Total	HVC	LVC	MVA	
	n (%)	n (%)	n (%)	*P* value	OR (95% CI)
RT duration (wk)					
≤7	938 (49)	715 (52)	223 (40)	<0.001	REF
>7	951 (49)	647 (47)	304 (54)	<0.001	0.31 (0.22‐0.43)
Unknown duration	41 (2)	6 (0)	35 (6)	<0.001	0.08 (0.02‐0.26)
Total RT dose					
<66/60 Gy	158 (8)	75 (6)	83 (15)	<0.001	REF
≥66/60 Gy	1673 (87)	1289 (94)	384 (68)	<0.001	4.12 (2.42‐7.02)
Unknown dose	99 (5)	4 (0)	95 (17)	0.001	0.11 (0.03‐0.39)
IMRT					
IMRT (−)	490 (25)	399 (29)	91 (16)	<0.001	REF
IMRT (+)	1038 (54)	880 (64)	158 (28)	0.007	0.59 (0.4‐0.87)
Unknown IMRT status	402 (21)	89 (7)	313 (56)	<0.001	0.05 (0.03‐0.08)
Chemotherapy					
Chemo (+)	748 (39)	421 (31)	327 (58)	<0.001	2.79 (1.91‐4.07)
Chemo (−)	1182 (61)	947 (69)	235 (42)		
DTI (d)					
≤45	971 (50)	715 (52)	256 (46)	0.447	—
46+	959 (50)	653 (48)	306 (54)		
Type of RT					
Definitive RT	1228 (64)	1018 (74)	210 (37)	<0.001	3.3 (2.1‐5.0)
Adjuvant RT	702 (36)	350 (26)	352 (63)		

95% CI, 95% confidence interval; DTI, diagnosis to treatment initiation; HVC, high‐volume center; HPV, human papillomavirus; IMRT, intensity‐modulated radiotherapy; LVC, low‐volume center; MVA, multivariate analysis; n, number of patients; OR, odds ratio; REF, reference variable; RT, radiation.

### 3.3 Stratification by definitive and adjuvant RT

#### 3.3.1 Definitive RT

**Figure 2:**
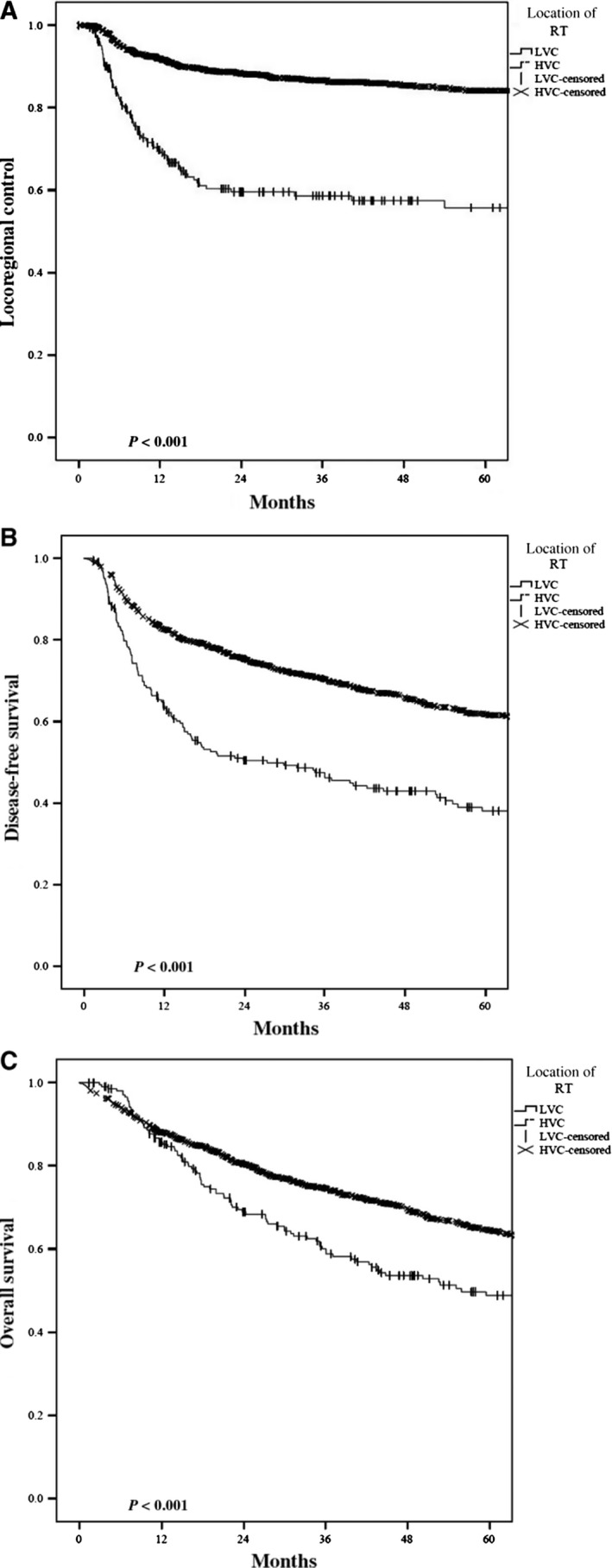
Kaplan‐Meier plots comparing High‐ vs Low‐volume center definitive radiation in regard to (A) locoregional control, (B) disease‐free survival, and (C) overall survival

For definitive RT patients, the overall 3‐year LRC, DFS, and OS were 82%, 67%, and 72% respectively. Radiotherapy at a HVC resulted in an absolute 3‐year LRC benefit of 28% (86.6% vs 58.6%, *P < *0.001) (Figure 2A). This benefit at 3 years was also seen with DFS (70.4% vs 47.4%) and OS (75% vs 60%) (*P < *0.001) (Figure 2B,C). On multivariate analysis, radiotherapy at a HVC remained independently associated with improvements in LRC (HR 3.94, 95%CI 2.69‐5.77, *P < *0.001), DFS (HR 1.77, 95% 1.43‐2.19, *P < *0.001), and OS (HR 1.45, 95%CI 1.12‐1.89, *P = *0.005) (Table 4).

**Table 4:** Predictors of locoregional control (LRC), disease‐free survival (DFS), and overall survival (OS) on multivariate analysis (MVA) of definitive and adjuvant RT cohorts

	LRC				DFS				OS			
	Def. RT		Adjuvant		Def. RT		Adjuvant		Def. RT		Adjuvant	
	MVA (*P*)	HR (95% CI)	MVA (*P*)	HR (95% CI)	MVA (*P*)	HR (95% CI)	MVA (*P*)	HR (95% CI)	MVA (*P*)	HR (95% CI)	MVA (*P*)	HR (95% CI)
Location of RT												
HVC	<0.001	0.24 (0.16‐0.36)	0.798	—	<0.001	0.5 (0.4‐0.62)	0.241		0.005	0.69 (0.53‐0.9)	0.54	
LVC												
Residence												
Elsewhere	0.41	—	0.621	—	<0.001	1.45 (1.2‐1.76)	0.137	—	<0.001	1.63 (1.34‐2)	0.046	1.29 (1.01‐1.65)
County of HVC												
Tobacco use												
Current	0.001	REF	0.324	—	<0.001	REF	0.084	REF	<0.001	REF	0.021	REF
Former	0.341	—			0.196	—	0.02	0.74 (0.58‐0.96)	0.025	0.79 (0.64‐0.97)	0.005	0.69 (0.53‐0.9)
Never	0.133	—			<0.001	0.46 (0.31‐0.69)	0.374	—	<0.001	0.41 (0.26‐0.66)	0.099	0.65 (0.39‐1.08)
Unknown use	<0.001	0.24 (0.12‐0.51)			<0.001	0.49 (0.35‐0.69)	0.833	—	<0.001	0.48 (0.34‐0.69)	0.724	
HPV/P16 status												
HPV (−)	0.013	REF	0.443	—	0.003	REF	0.493	—	<0.001	REF	0.687	—
HPV (+)	0.025	0.22 (0.06‐0.83)			0.051	0.41 (0.17‐1)			0.01	0.3 (0.12‐0.75)		
Unknown HPV status	0.441				0.525				0.601			
Primary site												
Oral cavity/LIPS	<0.001	REF	<0.001	REF	<0.001	REF	<0.001	REF	<0.001	REF	0.001	REF
Oropharynx	0.001	0.4 (0.23‐0.68)	<0.001	0.14 (0.05‐0.39)	<0.001	0.37 (0.26‐0.55)	<0.001	0.45 (0.3‐0.66)	<0.001	0.37 (0.25‐0.55)	<0.001	0.47 (0.31‐0.7)
Larynx	0.005	0.42 (0.23‐0.77)	0.001	0.36 (0.2‐0.64)	<0.001	0.43 (0.28‐0.64)	0.009	0.68 (0.51‐0.91)	<0.001	0.41 (0.27‐0.63)	0.028	0.72 (0.53‐0.96)
Hypopharynx	0.579	—	0.131	—	0.834	—	0.244	—	0.708	—	0.412	—
Nasopharynx	0.001	0.25 (0.11‐0.58)	0.98	—	<0.001	0.34 (0.21‐0.55)	0.923	—	<0.001	0.34 (0.2‐0.58)	0.925	—
Nasal cavity/sinus	0.748	—	0.132	—	0.052	0.53 (0.28‐1.01)	0.055	0.6 (0.35‐1.01)	0.002	0.34 (0.17‐0.68)	0.115	—
Salivary	0.332	—	0.17	—	0.692	—	0.872	—	0.112	—	0.716	—
Unknown primary	0.939	—	0.996	—	0.621	—	0.209	—	0.235	—	0.201	—
Tumor stage												
T1	<0.001	REF	0.356	—	<0.001	REF	0.217	—	<0.001	REF	0.387	—
T2	0.014	1.83 (1.13‐2.97)			<0.001	1.77 (1.33‐2.37)			<0.001	1.77 (1.29‐2.42)		
T3	0.001	2.33 (1.41‐3.87)			<0.001	2.28 (1.69‐3.08)			<0.001	2.6 (1.88‐3.59)		
T4	<0.001	4.81 (2.9‐7.95)			<0.001	4.33 (3.18‐5.88)			<0.001	4.82 (3.49‐6.66)		
T0/TX	0.942	—			0.82	—			0.821	—		
Nodal stage												
N0	0.181	—	0.004	—	<0.001	REF	<0.001	REF	<0.001	REF	<0.001	REF
N1	0.959		0.628	—	0.479	—	0.316	—	0.122	—	0.118	—
N2	0.926		0.002	1.87 (1.27‐2.76)	0.492		<0.001	2.01 (1.54‐2.62)	0.214		<0.001	2.18 (1.66‐2.87)
N3	0.063		0.957	—	<0.001	2.24 (1.57‐3.2)	0.04	2.42 (1.04‐5.63)	<0.001	2.55 (1.78‐3.66)	0.01	3.04 (1.3‐7.1)
Chemotherapy												
Chemo (+)	0.043	0.65 (0.43‐0.99)	0.854	—	0.733	—	0.039	1.29 (1.01‐1.64)	0.858	—	0.076	1.25 (0.98‐1.61)
Chemo (−)												
RT duration (wk)												
≤7	0.01	REF	0.65	—	0.111	—	0.002	REF	0.233	—	<0.001	—
>7	0.019	1.49 (1.07‐2.07)			0.037		0.001	1.43 (1.16‐1.76)			<0.001	1.48 (1.19‐1.84)
Unknown duration	0.119	REF			0.768		0.037	1.75 (1.03‐2.95)			0.003	2.21 (1.31‐3.72)
IMRT												
IMRT (−)	0.065	—	0.541		0.269		0.1		0.508		0.072	
IMRT (+)							0.031	0.75 (0.57‐0.97)			0.027	0.73 (0.55‐0.97)
Unknown IMRT status							0.262				0.525	
Total RT dose (NCCN)												
<60/66 Gy	0.447	—	0.596	—	0.518	—	0.419	—	0.005	REF	0.121	—
≥60/66 Gy									0.001	0.56 (0.4‐0.78)		
Unknown dose									0.046	0.57 (0.32‐0.99)		
DX to first treatment (d)												
46+	0.389	—	0.444	—	0.26	—	0.6	—	0.082	1.19 (0.98‐1.44)	0.743	—
≤45												
Age (y)	0.093		0.063		<0.001	1.03 (1.02‐1.04)	<0.001	1.02 (1.01‐1.03)	<0.001	1.04 (1.03‐1.05)	<0.001	1.03 (1.02‐1.04)
Location of surgery												
Outside hospital	—	—	0.339		—	—	0.772		—	—	0.509	
At our HVC	—	—			—	—			—	—		
Surgical margins												
SM (−)	—	—	0.03		—	—	0.012		—	—	0.059	
SM (+)	—	—	0.012	1.61 (1.11‐2.33)	—	—	0.008	1.4 (1.09‐1.8)	—	—	0.073	1.27 (0.98‐1.65)
Unknown SM status	—	—	0.463		—	—	0.254		—	—	0.195	
Surgery to port (wk)												
TTPORT ≤ 7	—	—	0.22		—	—	0.468		—	—	0.889	
TTPORT > 7	—	—			—	—			—	—		
Port package time (d)^a^												
<100	—	—	—		—	—	—		—	—	—	
≥100	—	—			—	—			—	—		
Unknown package time	—	—			—	—			—	—		

Def. RT, definitive RT; DFS, disease‐free survival; DTI, diagnosis to treatment initiation; HVC, high‐volume center; HPV, human papillomavirus; IMRT, intensity‐modulated radiotherapy; LRC, locoregional control; LVC, low‐volume center; MVA, multivariate analysis; OS, overall survival; PORT, postoperative radiation; REF, reference variable; RT, radiation; TTPORT, time to postoperative radiation.

a Not included on MVA.

Additional treatment characteristics associated with clinical outcomes include the following: IMRT use, RT duration, and RT dose on UVA. On multivariate analysis, extended RT duration >7 weeks (HR 1.46 95%CI 1.05‐2.02, *P = *0.023) and an RT dose <66 Gy (HR 1.89 95%CI 1.35‐2.65, *P < *0.001) predicted for a detriment in LRC and OS, respectively (Table 4). Treatment with IMRT was not an independent predictor of outcome on MVA. Additional patient and tumor characteristics that predicted for outcome on MVA include HPV status, primary site treated, tumor stage, nodal stage, and smoking status (Table 4).

The majority of definitive RT patients were treated with concurrent chemotherapy (77%), IMRT (61%), RT dose ≥66 Gy (90%), and with an RT duration >7 weeks (56%) (Table 5). On logistic regression multivariate analysis, RT at a HVC was less likely to have a prolonged RT duration >7 weeks (OR 0.49 95%C 0.31‐0.77, *P < *0.001) and was more likely to have RT dose ≥66 Gy (OR 2.36, 95%CI 1.08‐5.12, *P = *0.031). As we were unable to ascertain the exact RT treatment modality in many LVC patients, unknown IMRT status more often occurred in LVC‐RT (*P < *0.001). There was no difference in the use of chemotherapy (*P = *0.176) or delays to initiation of treatment (*P = *0.186) between LVC and HVC radiotherapy.

**Table 5:** Comparison of treatment characteristics of definitive RT cohort

Treatment characteristics	HVC	LVC	MVA	
	n (%)	n (%)	*P* value	OR (95% CI)
RT duration (wk)				
≤7	466 (46)	62 (30)	<0.001	REF
>7	546 (54)	136 (65)	<0.001	0.49 (0.31‐0.77)
Unknown duration	6 (1)	12 (6)	0.036	0.19 (0.04‐0.9)
Total RT dose				
<66/60 Gy	59 (6)	22 (11)	<0.001	REF
≥66/60 Gy	955 (94)	145 (69)	0.031	2.36 (1.08‐5.12)
Unknown dose	4 (0)	43 (21)	<0.001	0.11 (0.03‐0.47)
IMRT				
IMRT (−)	272 (27)	29 (14)	<0.001	REF
IMRT (+)	690 (68)	60 (29)	0.694	0.9 (0.53‐1.52)
Unknown IMRT status	56 (6)	121 (58)	<0.001	0.07 (0.04‐0.12)
Chemotherapy^a^				
Chemo (−)	232 (23)	57 (27)	—	—
Chemo (+)	786 (77)	153 (73)		
DTI (d)^a^				
≤45	497 (49)	92 (44)	—	—
46+	521 (51)	118 (56)		

95%CI, 95% confidence interval; DTI, diagnosis to treatment initiation; HVC, high‐volume center; HPV, human papillomavirus; IMRT, intensity‐modulated radiotherapy; LVC, low‐volume center; MVA, multivariate analysis; n, number of patients; OR, odds ratio; REF, reference variable; RT, radiation.

a Not included on MVA.

#### 3.3.2 Adjuvant RT

**Figure 3:**
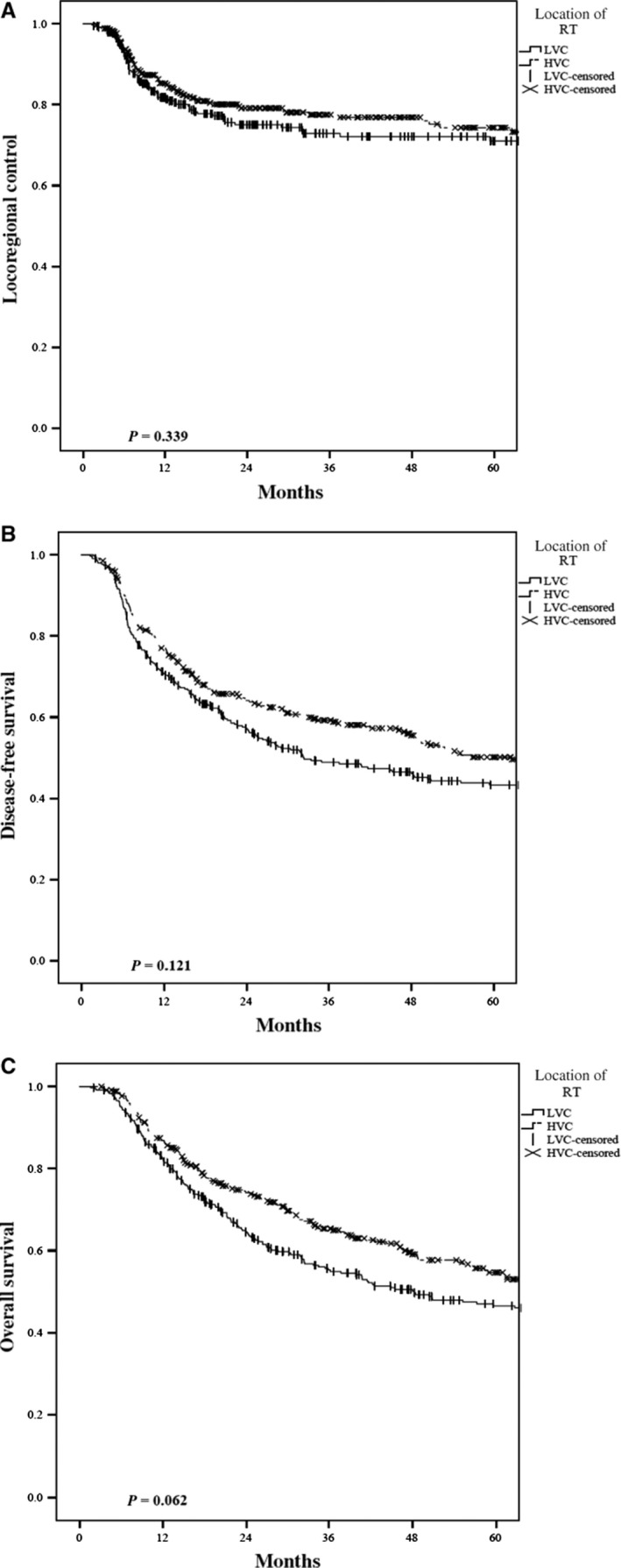
Kaplan‐Meier plots comparing High‐ vs Low‐volume center adjuvant radiation in regard to (A) locoregional control, (B) disease‐free survival, and (C) overall survival

For adjuvant RT patients, the overall 3‐year LRC, DFS, and OS were 75%, 54%, and 60%, respectively. In the adjuvant RT setting, HVC‐RT had no significant differences in 3‐year LRC (78% vs 73%, *P* = 0.339), or DFS (59% vs 49%, *P* = 0.121), and a trending OS benefit (65.3% vs 55.3%, *P* = 0.062) on UVA, when compared to LVC (Figure 3A‐C). Surgically associated treatment factors were included in the outcome analysis in the adjuvant RT setting. On MVA, positive surgical margins predicted for a detriment in LRC (HR 1.61, 95%CI 1.11‐2.33, *P* = 0.012), DFS (HR 1.4, 95%CI 1.09‐1.8, *P* = 0.008), and OS (HR 1.27, 95%CI 0.98‐1.65, *P* = 0.073). Time to postoperative RT (TTPORT>7 weeks), postoperative RT package time (≥100 days), and location of surgery did not independently predict for outcome (Table 4).

On MVA of the adjuvant RT subset, HVC‐RT was more likely to receive adequate RT dose (≥60 Gy, OR 9.86 CI95% 4.16‐23.4, *P* < 0.001), less likely to have prolonged RT duration (>7 weeks, OR 0.2 CI95% 0.12‐0.34, *P* < 0.001), and more likely receive concomitant chemotherapy (OR 3.09 CI95% 1.83‐5.2, *P* < 0.001), when compared to LVC (Table 6). HVC‐RT patients were less likely to have delays from surgery to the start of postoperative RT (TTPORT>7 weeks: OR 0.6 CI95% 0.37‐0.98, *P* = 0.039), less likely to have surgery at our tertiary referral center (OR 0.6 CI95% 0.37‐0.98, *P* = 0.039).

**Table 6:** Comparison of treatment characteristics of adjuvant RT cohort

Treatment characteristics	HVC	LVC	MVA	
	n (%)	n (%)	*P* value	OR (95% CI)
RT duration (wk)				
≤7	249 (71)	161 (46)	<0.001	REF
>7	101 (29)	168 (48)	<0.001	0.21 (0.12‐0.34)
Unknown duration	0 (0)	23 (7)	0.997	—
Total RT dose				
<66/60 Gy	16 (5)	61 (17)	<0.001	REF
≥66/60 Gy	334 (95)	239 (68)	<0.001	9.86 (4.16‐23.4)
Unknown dose	0 (0)	52 (15)	0.997	—
IMRT				
IMRT (−)	127 (36)	62 (18)	<0.001	REF
IMRT (+)	190 (54)	98 (28)	0.062	0.58 (0.32‐1.03)
Unknown IMRT status	33 (9)	192 (55)	<0.001	0.03 (0.02‐0.07)
Chemotherapy				
Chemo (−)	189 (54)	270 (77)	<0.001	3.09 (1.83‐5.2)
Chemo (+)	161 (46)	82 (23)		
DTI (d)				
≤45	218 (62)	164 (47)	0.218	—
46+	132 (38)	188 (53)		
Location of surgery				
Outside hospital	145 (41)	56 (16)	<0.001	0.4 (0.23‐0.72)
At ouR HVC	205 (59)	296 (84)		
Surgery to port (wk)				
TTPORT ≤ 7	187 (53.4)	154 (43.8)	0.039	0.6 (0.37‐0.98)
TTPORT > 7	153 (46.6)	198 (56.3)		
Package time (d)^a^				
<100	215 (61)	139 (40)	—	—
≥100	135 (39)	190 (54)		
Unknown package time	0 (0)	23 (7)		

95%CI, 95% confidence interval; DTI, diagnosis to treatment initiation; HVC, high‐volume center; HPV, human papillomavirus; IMRT, intensity‐modulated radiotherapy; LVC, low‐volume center; MVA, multivariate analysis; n, number of patients; OR, odds ratio; PORT, postoperative radiation; REF, reference variable; RT, radiation; TTPORT, time to postoperative radiation.

a Not included on MVA.

## 4 DISCUSSION

Recent evidence suggests head and neck cancer (HNC) patients treated with radiotherapy benefit from the institution's expertise,^6^, ^7^, ^9^, ^11^ attributed to both the experience of the physician^9^, ^19^, ^20^ and the facility.^8^, ^21^, ^22^ This expertise can result in higher adherence to nationally recognized treatment guidelines,^6^, ^7^, ^11^, ^12^ improved multidisciplinary care,^8^ and a decrease in RT breaks,^6^, ^11^ which are all potential contributors to patient outcome. In our study, all patients were evaluated at a HVC, potentially controlling for differences in patient workup, diagnosis, and access to care. Our evaluation found that HVC‐RT independently predicted for an outcome benefit in HNC. This improvement in LRC, DFS, and OS observed in HVC‐RT is especially important in the definitive setting, where RT plays the central treatment role and subsequently has a larger impact on outcome. Ideal treatment parameters (eg, RT duration and dose) were more likely to occur at a HVC. Even after taking dose and duration into account, HVC‐RT was independently prognostic for a LRC and DFS benefit, suggesting that there may be additional benefits with HVC‐RT that are difficult to account for in our analysis (eg, treatment volumes, target definition, nodal coverage, and patient management).

With the current trend in centralization of cancer care, it is important to identify specific characteristics that may lead patients to choose treatment at a LVC as opposed to a HVC. Patients in our study were 17 times more likely to undergo HVC‐RT if they lived in the HVC's county, which suggests that distance may be the primary influence of choosing RT location, a cause that has been previously described in the literature.^23^, ^24^, ^25^ HNC patients requiring definitive RT, as compared to those needing adjuvant RT, were >3 times more likely to undergo their RT at a HVC, which suggests that patients are more amenable to choosing a HVC for their weeks of RT when it is the primary component of their care. Other studies in surgical series have evaluated the relationship between patient travel distance, type of treatment facility, and outcomes.^26^ They found that despite the added burden of travel, patients who chose to receive treatment at a HVC had better outcomes.^26^ In addition, studies have reported that travel distance not only influences patient choice of treatment location but may also have an effect on their choice of treatment type.^23^ A recent study from Canada suggests that patients were more inclined to travel to a HVC once they understood the potential implications.^27^ Other studies suggest that even with the knowledge of improved outcomes at a HVC, a large proportion of patients are willing to undergo treatment at a LVC in order to minimize the travel burden.^28^ Further research in the specific socioeconomic trends of patients that choose LVCs despite having access to a HVC needs to be done to elucidate why these patient are willing to accept worse outcomes.

Although disparities in cancer care are well recognized, recent studies suggest that the current push for regionalization of cancer treatment can lead to an increase in the already present disparities gap.^29^ Recognizing distance as one of the main patient characteristics influencing choice of treatment facility therefore represents a potentially simple intervention that may impact the disparities gap. The identification of a volume‐outcome association and the centralization of cancer care further increase the importance of social work support in HVCs. In addition to educating patients regarding the volume‐outcome association, implementing social work strategies, such as access to transportation and lodging, may further help close the disparities gap as well as improve outcomes.

Previous studies evaluating radiation expertise in HNC, variably defined as academic centers (ACs), teaching facilities, or historically high‐accruing centers, have shown improvements in OS of 10%‐20%.^6^, ^7^, ^9^, ^11^, ^13^, ^19^, ^20^ For example, the Taiwanese National Health Insurance database was used to examine the relationship between radiation oncologist case volume and nasopharyngeal cancer (NPC) outcomes in Taiwan. Chien et al^20^ found an improved 5‐year OS associated with high‐volume radiation oncologists (77% vs 64%, *P = *0.0007). Lee et al^9^ showed a 10‐year OS benefit in NPC patients when treated by high‐volume radiation oncologists (75% vs 61%, *P < *0.001) on MVA, after adjusting for comorbidities, hospital level, and treatment modality. Neither study found a benefit based on level of hospital accreditation. This suggests a radiation oncologist's volume provides a survival advantage in NPC, likely due to improved radiotherapy planning quality and management for this complex disease.

A re‐analysis of the RTOG 0129 trial evaluated outcome based on facility accrual volume. Historically, high‐accruing centers (HHACs) were defined as the facilities in the top third of patient accrual. On MVA, HHACs resulted in an absolute 5‐year progression‐free survival and OS benefit of 19% (61.8% vs 42.7%, *P < *0.001) and 18% (69.1% vs 51%, *P = *0.002), respectively.^7^ This is consistent with the absolute 3‐year OS improvement of 15% seen in our study.

National Comprehensive Cancer Network (NCCN) guidelines emphasize that optimal patient management is best achieved when patients are treated by an experienced multidisciplinary team with a focus in HNC.^30^ Institutional expertise has been associated with improved adherence to nationally recognized treatment guidelines.^6^, ^7^, ^11^ In previous studies, ACs have RT doses consistent with NCCN guidelines (approximately 10% higher than non‐ACs), which the authors suggest is a contributing factor to AC's improved outcome.^6^, ^11^ Prolonged RT duration is most often linked to treatment breaks secondary to toxicity. Toxicity can be minimized by normal tissue avoidance during treatment planning, appropriate RT dosing and coverage, and adequate symptom management. RT duration >7 weeks has been associated with increased risk of local failure and death in HNCs treated with radiation, particularly radiation without concurrent chemotherapy.^31^ In the re‐analysis of RTOG 0129, high‐accruing centers had fewer protocol deviations (6% vs 18%, *P = *0.002), which may have contributed to 19% of their OS benefit.^7^ It has also been suggested that the higher rate of RT breaks in non‐ACs vs ACs (16.4% vs 0%, *P < *0.001) contributes to worse outcomes.^6^ RTOG 0129's high‐accruing centers were associated with a decrease in RT duration (47 vs 49 days, *P = *0.004). These findings are consistent with our study, where the HVC was more likely to give ≥66 Gy and complete RT without significant treatment breaks.

IMRT for HNC is significantly more complex than the 3D conformal RT used in RTOG 0129. For example, Hong et al^5^ evaluated the treatment design of 20 institutions with established HNC IMRT expertise. They found substantial variability in treatment volumes, target definition, prescription, nodal coverage, and use of chemotherapy. In a study with HNC treated primarily with IMRT, Lassig et al^13^ found an improved 5‐year survival rate in favor of ACs (53% vs 33%, *P < *0.001), despite no differences in radiation treatment dose or treatment breaks when compared to non‐ACs. There is also evidence that higher‐volume radiation oncologists can improve the HNC‐specific mortality of patients treated with IMRT.^19^

Overall, the strengths of our study include the following: large patient size, regional and diverse patient population that well represents the general public, modern treatment delivery, and a variety of primary sites. The limitations of this study include its’ retrospective nature, long time period, and missing data especially from outside centers (eg, HPV status, performance status, comorbidities, education, and income). These factors may influence patient outcome and access to care and travel (eg, access to care at a HVC). Although some socioeconomic factors were not available, previously described surrogates for disparity that were available were included in our analysis (eg, county of residence, race, Spanish ethnicity, and county of residence).

In conclusion, our study found that a HNC patient's choice for HVC radiotherapy is primarily influenced by their location of residence. We also found a direct association between travel distance, the patient's choice of treatment center, and patient outcomes. We report that HVC radiotherapy is associated with an absolute 3‐year benefit in LRC, DFS, and OS of 16%, 20%, and 15%, overall; with a larger absolute benefit in HNC treated with definitive RT (28%, 23%, and 15%). Although all patients had an initial evaluation at a HVC, some chose to pursue treatment at a LVC, which elucidates the impact of travel distance on the treatment facility chosen. In addition to identifying specific characteristics that may deter patients from receiving treatment at a HVC, in an effort to provide all patients the opportunity to receive quality care, HVCs should consider expanding social work services such as transportation and lodging during radiation in order to facilitate access to care.

## CONFLICT OF INTERESTS

None declared.
